# Tracking the global dispersal of a cosmopolitan insect pest, the peach potato aphid

**DOI:** 10.1186/1472-6785-9-13

**Published:** 2009-05-11

**Authors:** John T Margaritopoulos, Louise Kasprowicz, Gaynor L Malloch, Brian Fenton

**Affiliations:** 1 Department of Biochemistry-Biotechnology, University of Thessaly, Ploutonos 26, 41221 Larissa, Greece; 2 SCRI, Invergowrie, Dundee, DD2 5DA, UK

## Abstract

**Background:**

Global commerce and human transportation are responsible for the range expansion of various insect pests such as the plant sucking aphids. High resolution DNA markers provide the opportunity to examine the genetic structure of aphid populations, identify aphid genotypes and infer their evolutionary history and routes of expansion which is of value in developing management strategies. One of the most widespread aphid species is the peach-potato aphid *Myzus persicae*, which is considered as a serious pest on various crops in many parts of the world. The present study examined the genetic variation of this aphid at a world scale and then related this to distribution patterns. In particular, 197 aphid parthenogenetic lineages from around the world were analysed with six microsatellite loci.

**Results:**

Bayesian clustering and admixture analysis split the aphid genotypes into three genetic clusters: European *M. persicae persicae*, New Zealand *M. persicae persicae *and Global *M. persicae nicotianae*. This partition was supported by *F*_ST _and genetic distance analyses. The results showed two further points, a possible connection between genotypes found in the UK and New Zealand and globalization of *nicotianae *associated with colonisation of regions where tobacco is not cultivated. In addition, we report the presence of geographically widespread clones and for the first time the presence of a *nicotianae *genotype in the Old and New World. Lastly, heterozygote deficiency was detected in some sexual and asexual populations.

**Conclusion:**

The study revealed important genetic variation among the aphid populations we examined and this was partitioned according to region and host-plant. Clonal selection and gene flow between sexual and asexual lineages are important factors shaping the genetic structure of the aphid populations. In addition, the results reflected the globalization of two subspecies of *M. persicae *with successful clones being spread at various scales throughout the world. A subspecies appears to result from direct selection on tobacco plants. This information highlights the ultimate ability of a polyphagous aphid species to generate and maintain ecologically successful gene combinations through clonal propagation and the role of human transportation and global commerce for expanding their range.

## Background

*Myzus persicae *(Sulzer) (Hemiptera: Aphididae) is an exceptional species in many respects. It is extremely polyphagous, highly efficient as a plant-virus vector and one of the most widespread insect pests, as it has been recorded on all continents where crops are grown [1]. The species has a typical aphid annual cycle (cyclical parthenogenesis), i.e., a sexual generation on peach during winter and spring, alternating with many parthenogenetic (all female) generations during spring on peach and on various crop and non-crop annual plant hosts in summer and autumn. The sexual generation may be lost either totally (obligate parthenogenesis) or partially (functional parthenogenesis when a few sexual forms are produced). Genotypes with different reproductive strategies can occur sympatrically in peach growing areas where populations on summer crops consist of new recombinants that have migrated from peach and old clones that survived the previous winter(s) parthenogenetically on winter hosts. Their proportions depend on the availability of peach trees for the sexual phase and the severity of winter which mostly affects the parthenogenetical overwintering [2-4]. The plasticity in the mode of reproduction is a great biological advantage for *M. persicae*, because as a species, it is able to adapt to different climatic conditions in terms of day length and temperature [5]. Sexual reproduction provides the advantages of cold hardy eggs and new gene combinations in the subsequent generations. Asexual reproduction has the advantage of maintaining successful gene combinations and success in temperate regions where green bridges are available. The effect of reproductive strategy on the population structure of *M. persicae *has gained much attention recently. Studies conducted in Australia [4,6] and Europe [7,8] showed that clonal diversity was greatest in populations capable of a sexual phase compared to parthenogenetic ones. The most extreme case of reduced variability has been documented from Scotland, where the majority of the stable long-term population appears to consist of only three genotypes [9,10].

*Myzus persicae *exhibits strong selection with respect to host-plant adaptation on tobacco. Tobacco-feeding populations show consistent morphological differences from those on other crops, regardless of the mode of reproduction or origin [11-13]. The subspecies name, *Myzus persicae nicotianae*, has been given to the tobacco population [14] and genetic differences have also been reported between the specialist (*nicotianae*) and the generalist (*persicae*) subspecies [15]. The two taxa experience multifarious divergent selection, i.e., selection against cross-host migrants and their subsequent generations, which is crucial for the maintenance of host specialization [16,17]. In outdoor choice experiments with winged females, it has been shown that the two taxa have evolved an improved host recognition mechanism which is based on chemical cues perceived prior to the initiation of feeding [17]. Gene-flow between the two taxa is reduced due to differences in the mode of reproduction (asexual *vs*. sexual) or to the existence of prezygotic reproductive isolation mechanism in sexual populations [15].

*Myzus persicae *has developed multiple insecticide resistance mechanisms which have spread to many parts of the world due to aphid migration or to human transport activity [18]. The international trade in plants offers considerable potential for the widespread distribution of insect pests [19] and *M. persicae *is ideally suited for this. It is associated with many transportable crop hosts and its primary host, the peach tree, has been spread throughout the world. Knowledge of the inter-regional or inter-continental dispersal routes of *M. persicae *and the between region genetic variation will be helpful in elucidating several aspects of its ecology that offer explanations for the spread and persistence in heterogeneous environments of successful genotypes. This information could also help to predict the evolution and spread of insecticide resistance mechanisms at both regional and global scale, to locate sources of host-plant resistance or biological control agents and to make inferences about the relative fitness and persistence of the pest. To our knowledge no other study has attempted to examine the genetic variation of *M. persicae *or the spread of certain genotypes at such a global scale. Some studies have focused mainly on insecticide resistance mechanisms. For instance, a phylogenetic analysis of a fragment of the *para*-sodium channel gene flanking the *kdr *and *super-kdr *mutations in samples from various countries and continents suggested multiple independent origins of both mutations [20]. In other recent studies the genetics of *M. persicae *was examined using microsatellite DNA genotyping analysis, but not at a global scale, mostly in populations of the same or of two neighbouring countries [6-8,21]. In some cases certain asexual genotypes were widespread and found on herbaceous crops from year to year [8,21].

In this paper we studied the genetic variation of *M. persicae *at a global scale by examining worldwide samples. A particular aim was to understand the population structure and to identify the possible routes of global dispersal.

## Results

Microsatellite analysis was carried out using four independent runs of 96 samples per run. It was possible to include some of the same samples on all four runs. The accuracy of the capillary system for alleles known to be the same was generally 1 bp or less. The ABI capillary genotyper produced slightly different size calculations for some of the samples that had been analysed previously using older ABI systems. The results were tabulated and used as input for population genetic analysis programmes.

### Bayesian clustering analysis

The posterior probabilities (PPD) of the whole data set, which included all the 197 parthenogenetic lineages, were calculated with STRUCTURE software [22] for *K *values 1–15 without any prior population information. Following the pointers for choosing *K *provided in previous studies [22,23], the best solution for *K *proved to be 3 in four independent runs. There was a sharp increase of PPD values with *K *moving from 1 to 3. For *K *> 3 the gain of information is rather less and exhibits gradually lower values. A plateau appears to be reached at *K *= 3 (see Additional file 1) and the information brought by the fourth *K *cluster (and the following) is less important than the information brought by the former three. It seems that splitting the samples in three clusters represents the optimal subdivision of the data and avoids unjustified and less informative oversplitting.

**Figure 1:**
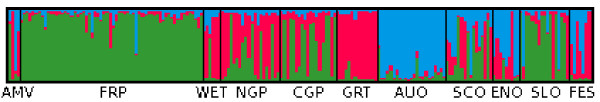
**Partition of genetic variation**. Admixture clustering plots of the 11 *Myzus persicae *samples examined. Number of clusters, *K *= 3; Custer 1 = green colour, Cluster 2 = red colour, Cluster 3 = blue. Each aphid lineage is represented as a vertical bar partitioned into *K *segments. The lengths of each segment are proportional to the estimated membership coefficients of the lineage in each of the three *K *clusters. Lineages of different samples are separated by black lines. AMV = America (first from peach in Argentina, second from weeds in Chile, the last two from pepper in Canada) (*n *= 4), FRP = France peach (four lineages from weeds) (*n *= 62), WET = Western Europe tobacco and pepper (the third lineage) (*n *= 6), NGP = northern Greece peach (*n *= 20), CGP = central eastern Greece peach (*n *= 19), GRT = Greece (northern and southern regions) tobacco (*n *= 14), AUO = New Zealand (plus two lineages from potato and peach in Australia) potato (*n *= 23), SCO = Scotland other than tobacco crops (*n *= 16), ENO = England other than tobacco crops (*n *= 9), SLO = Slovenia (the last lineage from Turkey) pepper and potato (*n *= 17), FES = Far East (first two from weeds from Sri Lanka, third from peach from Japan, fourth and fifth from radish and potato from Japan and the last two from tobacco from Japan) (*n *= 7). The samples from peach in northern Greece and Japan were from tobacco growing regions as was the sample from weeds in Chile. All the other non-tobacco samples were collected in non-tobacco growing regions.

DISTRUCT software [24] was used to create an admixture clustering plot of the 11 *M. persicae *predefined samples (Figure 1). Each aphid lineage is represented as a vertical bar partitioned into three coloured segments, the lengths of which are proportional to the estimated membership coefficients of the lineage in each of the three *K *genetic clusters defined by STRUCTURE. The four independent runs performed using STRUCTURE gave almost identical plots (result not shown). Cluster 1 contained almost all lineages from France and those from pepper and potato from Slovenia (samples FRP and SLO in Figure 1). One French lineage from weed showed a high membership coefficient to Cluster 3. Most of the peach lineages from eastern central Greece, some from peach in northern Greece and from UK crops (other than tobacco, termed here crops), one from peach in Argentina and one from pepper in Spain had high membership coefficients for Cluster 1.

Cluster 2 is characterized by the lineages collected from tobacco (sample WET except the third lineage, sample GRT and the last two lineages in sample FES in Figure 1). Most of the lineages from peach in northern Greece, where tobacco is widely cultivated, showed a high membership coefficient for Cluster 2. Lineages belonging to Cluster 2 were also found on crops in UK, Canada, Turkey and Slovenia, on weeds in Chile and on peach in central eastern Greece and Japan.

Cluster 3 is characterized by the lineages collected from potato in New Zealand and Australia (sample AUO in Figure 1). High membership coefficient to Cluster 3 was observed in various lineages from UK crops as well as in a few lineages from crops in Slovenia, Canada and Japan and weeds in Sri Lanka. Apart from the three samples France (FRP), Greece tobacco (GRT) and New Zealand/Australia (AUO) all the others were not pure and this is also demonstrated in their mean membership coefficients to the three clusters (Table 1).

**Table 1:** Average proportion of membership of each pre-defined aphid population in each of the 3 *K *genetic clusters derived from the Bayesian clustering analysis.

Populations^1^	Cluster 1	Cluster 2	Cluster 3	*n*^2^
FRP	0.889	0.052	0.060	62
WET	0.034	0.845	0.131	5
NGP	0.318	0.617	0.065	20
CGP	0.547	0.407	0.046	19
GRT	0.055	0.910	0.035	14
AUO	0.084	0.040	0.876	23
SCO	0.355	0.323	0.323	16
ENO	0.071	0.314	0.616	9
SLO	0.669	0.185	0.146	16

^1^FRP = France peach plus four lineages from weeds; WET = Western Europe tobacco, NGP = northern Greece peach, CGP = central eastern Greece peach, GRT = Greece (northern and southern areas) tobacco, AUO = New Zealand potato plus two lineages from Australia from potato and peach, SCO = Scotland other than tobacco crops, ENO = England other than tobacco crops, SLO = Slovenia pepper and potato. ^2^*n *= number of lineages examined.

### Allele frequencies, Hardy-Weinberg equilibrium and linkage disequilibrium

A moderate to high allelic diversity was found in the seven populations analysed. Apart from the highly polymorphic locus M49 (10–21 alleles) the number of alleles per locus and population ranged from 3 to 15 (> 7 in 51% of the cases) in the other five loci. The average over all loci and populations was 9.5 alleles/locus (for allele sizes see Additional file 2). Mean allelic richness (*R*s) ranged among populations from 7.2 to 11.8. Mean observed heterozygosity over all loci ranged from 0.623 to 0.855, with no significant differences among populations (*P *= 0.542). The UK population had the largest values for every diversity indicator (Table 2). Significant single locus deviation from HW equilibrium was observed in 8 out of the 56 tests. The loci which showed deviations were M35, M40, M49, M63 and myz9. In all cases the deviation was associated with a positive *F*_IS _value, i.e., a heterozygote deficiency. The multilocus test showed significant positive *F*_IS _values in four out of the seven populations examined (Table 3). Significant linkage disequilibrium was observed in 14 out of the 105 locus pairs examined.

**Table 2:** Genetic diversity indices for the *Myzus persicae *populations

Populations^1^	M49	M63	M86	M35	M40	myz9	All
FRP	16^2^	11	12	7	6	9	10.2
	10.4	7.6	9.0	4.3	5.0	7.1	7.2
	0.806	0.806	0.710	0.452	0.677	0.694	0.691
	0.864	0.794	0.839	0.449	0.764	0.813	0.754
NGP	10	7	9	6	4	9	7.5
	9.5	6.9	8.5	6.0	3.8	8.7	7.2
	0.900	0.700	0.800	0.850	0.350	0.800	0.733
	0.865	0.810	0.847	0.763	0.512	0.855	0.775
CGP	13	5	11	7	4	6	7.7
	12.3	5.0	10.4	7.0	4.0	5.7	7.4
	0.684	0.684	0.842	0.684	0.474	0.526	0.649
	0.861	0.644	0.852	0.797	0.687	0.713	0.759
EUT	11	7	10	7	3	12	8.3
	10.6	6.7	9.3	6.8	2.8	11.6	8.0
	0.842	0.579	0.789	0.474	0.158	0.895	0.623
	0.903	0.734	0.856	0.826	0.152	0.922	0.732
AUO	11	9	11	6	6	10	8.8
	9.3	8.3	9.9	5.7	5.7	8.4	7.9
	0.652	0.783	0.913	0.783	0.913	0.826	0.812
	0.807	0.776	0.849	0.757	0.817	0.796	0.800
UKO	21	14	14	10	9	15	13.8
	16.8	12.2	11.8	9.0	8.0	13.2	11.8
	0.913	0.913	0.957	0.609	0.783	0.957	0.855
	0.923	0.895	0.898	0.831	0.787	0.916	0.875
SLO	14	8	10	7	7	9	10.0
	14	8	10	7	7	9	10.0
	0.875	0.563	0.688	0.625	0.688	0.688	0.688
	0.909	0.800	0.833	0.683	0.756	0.752	0.789

FRP*	16	11	12	6	6	9	10.2
	10.5	7.8	8.7	4.1	5.1	6.9	5.4
	0.793	0.845	0.707	0.448	0.655	0.690	0.690
	0.858	0.805	0.834	0.432	0.763	0.806	0.750
NZO	9	9	11	6	6	8	8.2
	8.2	8.5	10.0	5.8	5.8	7.2	7.6
	0.619	0.810	0.905	0.810	0.952	0.810	0.818
	0.779	0.768	0.849	0.764	0.821	0.783	0.794

^1^FRP = France peach plus four lineages from weeds; NGP = northern Greece peach, CGP = central eastern Greece peach, EUT = western Europe and Greece tobacco, AUO = New Zealand potato plus two lineages from Australia from potato and peach, UKO = UK other than tobacco crops, SLO = Slovenia pepper and potato. FRP* = refers to FRP without the four lineages from weeds. NZO = refers to AUO without the two lineages from Australia. ^2^First line number of alleles, second line allelic richness (number of alleles independent of sample size, i.e., based on minimum sample size of 16 individuals), third line heterozygosity observed and fourth line heterozygosity expected.

**Table 3:** Single and multilocus probability tests for deviations from Hardy-Weinberg equilibrium and *F*_IS _values.

Populations^1^	*n*^2^	M49	M63	M86	M35	M40	myz9	Overall
FRP	62	+0.067 NS/NS^3^	-0.016 NS/NS	+0.155 NS/NS	-0.006 NS/NS	+0.115 0.009/NS	+0.148 0.022/NS	+0.084 0.004/NS
NGP	20	-0.041 NS/NS	+0.139 NS/NS	+0.057 NS/NS	-0.118 NS/NS	+0.321 NS/NS	+0.066 0.044/NS	+0.056 NS/NS
CGP	19	+0.209 NS/NS	-0.064 NS/NS	+0.012 NS/NS	+0.144 NS/NS	+0.316 0.041/NS	+0.267 0.034/NS	+0.148 0.0158/NS
EUT	19	+0.069 NS/NS	+0.216 NS/NS	+0.080 NS/NS	+0.434 0.002/NS	-0.038 NS/NS	+0.030 NS/NS	+0.153 0.022/NS
AUO	23	+0.195 NS/NS	-0.009 NS/NS	-0.077 NS/NS	-0.034 NS/NS	-0.120 NS/NS	-0.039 NS/NS	-0.014 NS/NS
UKO	23	+0.011 NS/NS	-0.021 NS/NS	-0.067 NS/NS	+0.272 0.003/NS	+0.006 NS/NS	-0.045 NS/NS	+0.023 NS/NS
SLO	16	+0.039 NS/NS	+0.304 0.008/NS	+0.179 NS/NS	+0.088 0.020/NS	+0.093 NS/NS	+0.088 NS/NS	+0.132 0.004/NS

FRP*	58	+0.076 NS/NS	-0.050 NS/NS	+0.153 NS/NS	-0.039 NS/NS	+0.143 0.006/NS	+0.145 NS/NS	+0.081 0.023/NS
NZO	21	+0.210 NS/NS	-0.056 NS/NS	-0.067 NS/NS	-0.061 NS/NS	-0.164 NS/NS	-0.035 NS/NS	-0.030 NS/NS

^1^FRP = France peach plus four lineages from weeds; NGP = northern Greece peach, CGP = central eastern Greece peach, EUT = western Europe and Greece tobacco, AUO = New Zealand potato plus two lineages from Australia from potato and peach, UKO = UK other than tobacco crops, SLO = Slovenia pepper and potato. FRP* = refers to FRP without the four lineages from weeds. NZO = refers to AUO without the two lineages from Australia. ^2^*n *= number of genotypes examined. ^3^Probabilities for Heterozygote Deficit/Heterozygote Excess, NS = non significant.

### F_*ST *_and genetic distance analysis

**Figure 2:**
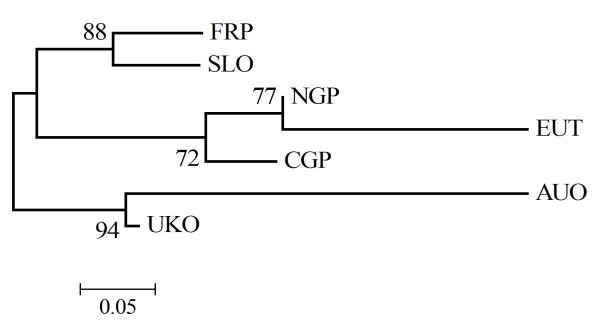
**Phylogeny of *Myzus persicae *populations**. Neighbour joining tree based on shared allele distances among seven *Myzus persicae *populations. Numbers denote bootstrap percentages (from 1000 resamplings). FRP = France peach plus four lineages from weeds; NGP = northern Greece peach, CGP = central eastern Greece peach, EUT = Western Europe and Greece tobacco, AUO = New Zealand potato (plus two lineages from Australia from potato and peach, UK = UK other than tobacco crops, SLO = Slovenia potato and pepper.

The pairwise multilocus *F*_ST _analysis revealed important interpopulation variation and the global *F*_ST _value was 0.086. Relatively high values were observed in comparisons between most of the European populations and those from Australasia. High values were also observed in most of the comparisons between the tobacco population from Europe (EUT) and those from tobacco-free regions (Europe: FRP, EUO, CGP; New Zealand/Australia: AUO). However, the peach population from a tobacco-growing region in northern Greece (NGP) showed mostly low *F*_ST _values when compared with the former two sample categories, suggesting similarity to both (Table 4). The UK population showed low or moderate *F*_ST _values in the comparisons with each of the other regions including Australasia suggesting it has links with all the major populations. The spatial population subdivision was also supported with the single-locus test for allelic differentiation as all pairwise comparisons were significant (Table 4). The NJ tree based on the DAS genetic distance (Figure 2) resulted in two major clusters. The first cluster contained the samples from New Zealand/Australia (AUO) and that from UK (UKO). In the second cluster tobacco lineages (sample EUT) and those from peach from the tobacco-growing region in northern Greece (sample NGP) formed a distinct group separated from the remaining samples.

**Table 4:** Multilocus *F*_ST _pairwise values and probabilities for allelic differentiation

	FRP	NGP	CGP	EUT	AUO	UKO	SLO
FRP	-	*	*	*	*	*	*
NGP	0.070	-	*	*	*	*	*
CGP	0.061	0.043	-	*	*	*	*
EUT	0.154	0.036	0.082	-	*	*	*
AUO	0.116	0.118	0.140	0.167	-	*	*
UKO	0.065	0.045	0.070	0.075	0.053	-	*
SLO	0.036	0.073	0.057	0.146	0.118	0.036	0.000

The numbers below the diagonal are the multilocus *F*_ST _values. Above the diagonal are the probabilities for the absence of allelic differentiation between populations are given (*P < 0.001). FRP = France peach plus four lineages from weeds; NGP = northern Greece peach, CGP = central eastern Greece peach, EUT = Western Europe and Greece tobacco, AUO = New Zealand potato plus two lineages from Australia from potato and peach, UKO = UK other than tobacco crops, SLO = Slovenia pepper and potato.

## Discussion

The present study has revealed a broadly heterogeneous genetic structure of *M. persicae *at a global scale as evidenced by high allelic differentiation and relatively high *F*_ST _values between certain populations, and the partitioning of genetic variation by the Bayesian clustering analysis. The observed genetic variation can be attributed to mode of reproduction, host-plant adaptation, differences between regions and dispersal.

### Genetic diversity and reproductive mode

A moderate to high genic diversity was found at the intrapopulation level, as well as cases of both single and multi locus deviations from HW equilibrium which was associated with heterozygote deficiency. The populations on peach, which are expected to contain only or mostly cyclical parthenogenetic genotypes, showed HW deviations in one or both of M40 and myz9 loci. In the populations from herbaceous hosts, which probably consist of a mixture of obligate/functional and cyclical parthenogens, some heterozygote deficiency was observed in M35, M49 and M63 loci. Heterozygote deficiency in microsatellite loci appears to be common among aphid species (*S. avenae*, France [25], *R. padi*, France [26]) including *M. persicae *(France [7,21], Australia [4] and Greece [15]) and it has been recorded in both sexual and asexual populations. However, some studies have found less deviation from HW equilibrium in sexual populations (*M. persicae*, Australia [6] and *M. persicae*, Greece [15]). In populations found on secondary hosts, HW deviation might be expected due to the presence of asexual lineages. Asexuality with strong clonal selection is likely to cause deviations from HW equilibrium in polymorphic loci, such as microsatellite markers, via hitch-hiking and evolution in clonal lineages. The direct effects of local clonal propagation were mediated in the current study by removing clone duplicates. In some cases, e.g., hI lineages of *Rhopalosiphum padi *(L.) in France [27] and *M. persicae *in Victoria Australia [4], a heterozygote excess has been found in asexual lineages. In *R. padi *this excess is attributed either to ancient loss of sexuality and the consequence of accumulated mutations or to a hybrid origin. In other cases, however, asexual lineages showed heterozygote deficiency and heterozygosity levels close to that of their sexual counterparts (hII *R. padi *lineages in France [27] and *M. persicae *in France [7]). This has been associated with a recent loss of sexuality and the time has not been sufficient to allow accumulation of mutations in asexual lineages. It has also been suggested that gene flow between sexual and asexual functional parthenogens producing males may be sufficient to prevent differences in heterozygosity accumulating between reproductive modes. Previous studies have discussed reasons why *R. padi *[26] and *M. persicae *[21] sexual populations show homozygous excess (selection, clonal expansion, Wahlund effect, inbreeding and other population effects). All of these studies concluded that null alleles were not responsible for the effects as these would have been detected during the scoring process. Wahlund effect of sampling from distinct gene pools in the same population may contribute to the homozygote excess at least in some populations that have been examined here. In support of this, the Bayesian analysis showed that some populations contain members of more than one genetic cluster.

### Partitioning of genetic variation – host-plant and region

In general, the high *F*_ST _values obtained in pairwise population comparisons and the estimated overall value (0.086) are among the highest reported in an aphid species using microsatellite markers [25,26,28] and in the same order as those reported in previous studies for *M. persicae *populations from Europe [7,15] and Australia [6].

The Bayesian clustering and admixture analysis partitioned the genetic variation into three clusters, European (1), tobacco (2) and Australasian populations (3). Clusters 1 and 3 correspond to the generalist *M. persicae persicae *while Cluster 2 corresponds to the tobacco-adapted subspecies *M. persicae nicotianae*. Cluster members are spread over all continents and in most of the countries from which populations have been examined. These results support the hypothesis that the globalization of agriculture will have an immediate impact on the evolution of pest populations. Previous studies have provided more direct evidence of this through the spread of obligate/functional parthenogenetic genotypes (see detailed discussion below). In addition to anthropogenic activity, *M. persicae *populations will be influenced by natural mating and biological processes according to geographical region (Australasia *vs*. Southern Europe) and to tobacco adaptation, i.e., *nicotianae vs*. *persicae *(tobacco *vs*. other crops, tobacco or peach in tobacco regions *vs*. peach in non tobacco regions). In general, the proportion of membership for the tobacco Cluster 2 was greater in tobacco-growing areas. In addition, the genetic distance and the *F*_ST _analyses supported the separation of the tobacco aphid populations as well as the regional population structure of *persicae*. It is worth noting that the separation between the peach population from eastern central Greece and the equivalent from northern Greece, as revealed by *F*_ST _and Bayesian analyses (membership coefficient of CGP to European *persicae*: 0.55), was not as strong as observed in a previous paper (membership coefficient of the equivalent peach population to *persicae *cluster: 0.78, [15]). A possible explanation is that the two peach samples from Greece examined here (NGP and CGP) were a mixture of genotypes of both subspecies at a different ratio according to the region. An influx of the tobacco aphids (Cluster 2) into the peach orchards of eastern central Greece associated with Cluster 1 and the converse in peach orchards in northern Greece could be an explanation.

### Tobacco-adapted lineages

Our results suggest that certain alleles are associated with the genotypes of *M. persicae *feeding on tobacco. This can be considered as defining a tobacco adapted aphid 'genome' perhaps encoding a series of important enzyme variants for this specialisation as a result of continuous selection on this plant. This phenomenon is associated with the taxon *M. persicae nicotianae *and according to our results it is widespread and appears to have moved into countries where tobacco is not cultivated (UK and Slovenia) or the cultivation is limited (Canada). It is not surprising to find *nicotianae *on other crops, since it is able to colonize and reproduce on various herbaceous hosts within the vast host range of *M. persicae *s.l. [16,29]. It is likely that the source of the UK *nicotianae *genotypes is Europe. Studies using the European suction trap network [30] have shown that *M. persicae *s.l. in Europe can migrate over southern England. Evidence that supports the continental origin of UK *nicotianae *is the identification in this study of a red *nicotianae *genotype found in the UK and also a tobacco region in southern Greece (and in Bulgaria, Fenton unpublished data). We also noticed that none of the *M. persicae *genotypes sampled historically in the UK (e.g., C, D, E, I, J, L in Fenton et al. [21,31], are amongst the tobacco Cluster 2 aphids. This suggests that *nicotianae *has arrived rather recently in UK. The ability of the tobacco aphid to colonise new territories, even if its optimal host is not present, has interesting evolutionary implications. In addition to marker differences, it also differs physically from *persicae*. Generally a red *nicotianae *colour morph predominates in various parts of the world [11] and red colour populations have been associated with a complete or partial loss of sexuality [3,32]. The red colour morph might have ecological advantages such as absorption of solar radiation [33] and lower choice selection by parasitoids [34]. The red form present on tobacco plants in North America may be more resistant to organophosphorus insecticides than the green form [35]. It has also been found that the red form of *M. persicae *s.l. mostly has an A1,3 autosomal translocation, which is linked to the E4-based resistance mechanism, whereas the same translocation only occasionally appears in the green form [11,36]. Lastly, parthenogenetic lineages of the red form of *M. persicae *s.l. have shown better performance on tobacco plants than green ones [37]. It has been hypothesised that adaptation to tobacco arose as a single evolutionary event in sexual populations, probably in East Asia where *nicotianae *was first reported as a pest [13,38]. The tobacco-adapted population then established as permanently asexual populations in various regions. In some temperate regions the availability of peach favoured the return to a yearly sexual generation. The Bayesian clustering and admixture analyses in the present study revealed a genetic similarity of the *nicotianae *genotypes which strengthens the hypothesis that the adaptation to tobacco was a single evolutionary event.

### The UK population and a potential link with Australasia

The UK population contained only 23 genotypes. Despite this it was the most diverse by every measurement. Approximately one third of the UK lineages belonged to each of the three Clusters. Given that peach is not openly cultivated in the UK, recent asexual populations appear to mostly develop from successive waves of colonising clones [9]. Surprisingly, unlike the rest of the European populations, elements of the UK population were most like the Australasian population. Taking into account the admixture clustering plot, it seems that the UK is a good candidate as a source of exchange with the gene pool of the Australasian (mainly New Zealand) aphids. The earliest introductions of exogenous aphids to Australia and New Zealand were likely to be associated with settlers from Europe, especially from the UK. In support of this, previous studies revealed many common microsatellite alleles between *M. persicae *genotypes from Australia [6] and Europe [39]. Europe does also seem to be the origin of other non-indigenous Australasian aphid pest species such as *Elatobium abietinum *(Walker) (Hemiptera: Aphididae) [40]. In the present study, parameters were similar or higher when the New Zealand population were compared with European populations. Moderate or high genetic diversity has also been reported in previous New Zealand [41] and Australian studies [4,6]. Theoretical and empirical work suggests a general pattern of loss of genetic diversity during colonization [42,43]; this is because emigrant populations are serially bottlenecked [44,45]. The substantial genetic variation observed in New Zealand and Australian *M. persicae *suggests that the species has not been bottlenecked and this could be attributed to sexual reproduction [41,46] and the time that it has been there. In Australia the species was first recorded in 1910 [47], although it is believed to have existed there since at least 1893. This period is adequate for the mutation of new microsatellite length alleles in asexual lineages [48] and for sexual reproduction to give rise to diversified genotypes. In the aphid *Schizaphis graminum *(Rondani) (Hemiptera: Aphididae), which was introduced in the USA in the 1880s, sexual reproduction was considered as the main reason for the high diversity observed [49]. The UK *M. persicae *population is believed to lack holocyclic forms and therefore restoration of the full sexual cycle through mutation or though breeding between clones with partial loss of sexual reproduction (functional parthenogenesis) [50] may have been required. Nevertheless, introduction of sexual clones cannot be excluded. Another factor is possible multiple introductions of *M. persicae *in New Zealand. Van Toor et al [41] reported that clones NZ2 and NZ3 appeared to be introduced as they contained many unique alleles when compared to the remaining NZ population. Additional support for the existence of multiple introductions was found when NZ3 was recognised as being a common asexual *M. persicae *clone found in Scotland (clone D in Fenton et al. [31]). Exchange of genotypes between New Zealand and Australia could also occur as demonstrated for *Sitobion *genotypes [51]. The two Australian lineages examined in the current study had 17 of the 21 alleles recorded in common with the New Zealand population suggesting a recent common origin.

### Clone dispersal

The present study revealed some genotypes that were sampled many miles apart in different countries some of which had been identified before, e.g., Clones B (UK and Turkey) and D (UK and New Zealand) [31,41], and others we identified for the first time in the current study e.g., Clone M in UK and Slovenia; a genotype found in France and Greece, another in UK, Greece and Bulgaria and another in southern Greece and Slovenia. These studies have suggested that widespread clones appear to occur as a result of selection for insecticide resistance in agriculture [9,31,41,52]. We have examined a relatively small number of individuals in the *M. persicae *population, yet we have detected these clones. This suggests that the number of successful insecticide resistant genotypes is still relatively limited, despite the possibility of resistance genes combining into more and more genotypes in sexual populations every year. In addition to the spread of resistant clones, it has also been found that asexual tobacco aphid lineages have spread between neighbouring countries such as Greece and Italy [8] and in the current study a widespread *nicotianae *lineage was found in southern Greece and Slovenia and another in Greece, UK and Bulgaria. We also report here that a distinct tobacco lineage has been found in Greece and Chile. A previous study found only one microsatellite genotype of the tobacco aphid in Chile and it seems likely that the lineage we have identified is the same as that reported by Fuentes-Contreras et al. [32]. These studies suggest an old world origin of southern American *nicotianae *as the subspecies exhibits genotypic variation in Europe [8], but none in Chile [32]. During the last decade several studies have revealed that the spread of certain genotypes over distant geographical areas is a common phenomenon among aphid species (e.g., *Sitobion *[51], *S. avenae *[25,28,53,54]) including *M. persicae *[4,8,31,41]. The rapid spread of the *M. persicae *s.l. lineages in different countries and continents should be attributed mostly to human transport and commerce. While winged aphids may be transported very rapidly over great distances by low level-jet streams [55] other studies have found that particular genotypes remain localised [10]. The widespread lineages reported in the current work probably represent asexual genotypes reproducing parthenogenetically all-year-round. This trait enables them to spread because their reproduction will not be altered by temperature, day length or the requirement for peaches to complete their life cycle. These clones might represent 'general-purpose genotypes' [56] with broad ecological tolerance, which predominate in fluctuating environments through selection, although anthropogenic activities, e.g., insecticide selection pressure might also be involved [9,52].

## Conclusion

**Figure 3:**
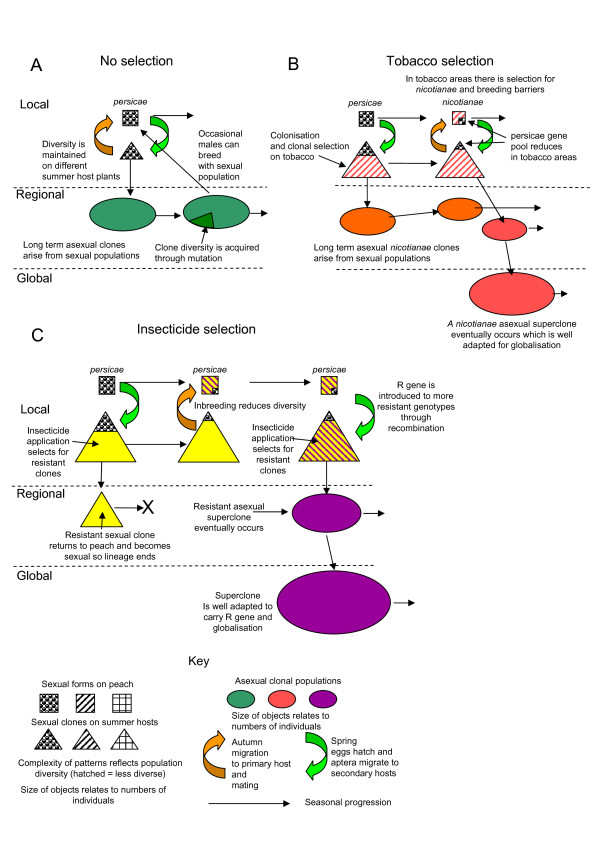
**Processes affecting population structure in *Myzus persicae***. The figure illustrates processes that influence the genetic structure of the aphid population at local, regional and global scale, with emphasis on selection due to host-plant and insecticide pressure. A. Represents the basic population of *M. persicae *living in and around peach trees and orchards. The emerging spring population is diverse and this diversity is maintained because no lineage dominates during the summer months and gametes have equal opportunities for mating at the end of the return migratory phase. These populations can produce asexual clones from time to time. B. Represents the situation where tobacco cultivation is close to peach trees. Tobacco selects for particular gene combinations and this in turn carries alleles associated with these genes. Over time, tobacco selection pressure has generated breeding barriers and a distinct aphid form. The tobacco form shares many characteristics of the main group being capable of growing on a range of host plants and globalisation. C. Represents the process that occurs when an insecticide resistance allele occurs in a population for the first time. There will be an immediate advantage for individuals in a clone carrying this resistance allele and within one season their numbers would increase rapidly. At the end of that season a sexual lineage will return to peaches in vast numbers, where it will mainly inbreed. Over time two events are likely, that the resistance allele will spread into more genotypes and that some of these genotypes will become asexual clones and then superclones capable of globalisation.

The present study is the first attempt to elucidate the pattern of global genetic variation of *M. persicae *s.l. using high resolution DNA markers. Figure 3 is a representation of some of the processes that influence population structure. The populations of *M. persicae *in and around peach orchards exhibit considerable genetic diversity and in some cases population parameters are close to those of sexually reproducing insects (Figure 3a). Geographical separation does create discrete gene pools as exhibited by the separation of New Zealand and European sexual populations. Sympatric speciation has generated a tobacco race of *M. persicae *and this process could clearly have been aided by agriculture with monocultures selecting tobacco adapted genotypes (Figure 3b). In addition to selection by tobacco, selection by insecticides is likely to have played a role in determining population structure. In the early stages of the evolution of resistant genotypes inbreeding could occur, reducing population diversity (Figure 3c). In all of these cases, any well adapted genotype could bypass sexual reproduction to become clone. The most successful of these asexual lines will become superclones spreading naturally as well as being aided by commercial activities (Figure 3). Long lived superclones will accumulate mutations. Examples of these superclones can be found in both *nicotianae *and *persicae *lineages. Evidence was reported of movement of the tobacco aphid from Europe to S. America and the spread of some *persicae *lineages to geographically distant regions (Europe to New Zealand). UK populations consist of asexual clones originating from diverse sources. This information highlights the ultimate ability of polyphagous aphids to adapt to different environmental conditions and the role of commerce in the globalization of insect pests.

## Methods

### Aphid samples

The data set consisted of 197 aphid lineages collected from 14 countries in four continents mainly from peach and tobacco but other herbaceous hosts were included (Table 5). To obtain these unique genotypes, thousands of individuals had been sampled and analysed in the various study areas over a ten year period. The samples from peach in northern Greece and Japan were from tobacco growing regions as was the sample from weeds in Chile. All the other samples were collected in non-tobacco growing regions [see 21 (France), 41 (NZ), 10(UK)]. The samples from herbaceous crops from UK, Slovenia and southern Greece were from non peach-growing areas and they should consist mostly of asexual lineages. Most lineages were reared parthenogenetically under laboratory conditions and specimens from each lineage were kept at -80°C or in tubes filled with absolute ethanol until microsatellite analysis. Some of the samples consisted of a single aphid collected directly from the source tree or plant and stored as above.

**Table 5:** *Myzus persicae *lineages used in the study.

Region	Collection Year (month)	Locality	Crop	Colour		Total
					
				Green	Red	
Canada	2005 (iv)	-	Pepper	1	1	2
Argentina	1993 (xii)	-	Peach	1		1
Chile	2005 (ix)	El molle	Weed		1	1
UK (Scotland)	1995, 2001–04	Various	B sprt, cabbage, oilseed rape, potato	13	3	16
UK (England)	1987, 1991, 1997, 1999, 2002–03	Lincs, Yorks, Kent Herts, Suffolk, Cambs, Norfolk	B sprt, cabbage, oilseed rape, sugar beet, potato	7	2	9
North Greece	2006 (vi)	Meliki	Peach	20	0	20
North Greece	2006 (vii, viii)	Meliki	Tobacco	8	1	9
Eastern central Greece	2006 (vi)	Lechonia	Peach	19		19
South Greece	2005 (vi)	Naphplion	Tobacco	1	4	5
France	2001, 2003	Bellegarde, Nimes	Peach	53	5	58
France	2001	Nimes	Weeds	4		4
Germany	1999		Tobacco		2	2
Slovenia	2006	Ljubljana, Krosko	Pepper, potato	16		16
Spain	1999 (vii)	Madrid	Tobacco	1	2	3
Spain	1992 (vii)	-	Pepper	1		1
Turkey	1998	-	Pepper	1	0	1
Japan	1993 (v)	Kyoto	Peach	1		1
Japan	1982 (vii), 1997 (vii)	Kyoto, Funehiki	tobacco		2	2
Japan	1995 (V), 2001 (x)	Kyoto	Radish, potato	1	1	2
Sri Lanka	2006	Sri Lanka	Weed	2		2
Australia	2005	-	Peach	1		1
Australia	2005	-	Potato	1		1
New Zealand	2005	Lincoln, Christchurch, Dorie, Pukekohe, Rakaia, Ashburton, Pukekohe	Potato	21		21
			Total	173	24	197

In some cases the numbers of individuals representing an area were low and in these cases samples collected from the same region or continent and host, as well as data between years, were pooled for some of the analyses (i.e., genetic diversity indices, Hardy-Weinberg equilibrium, linkage disequilibrium, *F*_ST _and genetic distance analysis). Samples from crops other than tobacco were also combined (termed here 'crops'). Both unique and multicopy genotypes were included in the analysis, but with only one copy of each multicopy genotype per population in order to avoid artificial deviations from the Hardy-Weinberg and linkage equilibria within populations and distorted estimates of allele frequencies [57].

### DNA extraction and microsatellite genotyping

Details on DNA extraction, microsatellite loci amplification, analysis and visualization are present in a previous paper [58]. Six microsatellite loci; M35, M40, M49, M63, M86 and myz9 [59], were chosen on the basis of their resolution (based on allele numbers of 12, 11, 35, 19, 21 and 18, respectively, giving 2.43 × 10^13 ^possible combinations). Many of the lineages had been analysed in earlier work [e.g., [21]]. Therefore, to eliminate any doubt over allele size scoring due to technical modifications, the entire collection of genotypes was analysed again for this study using exactly the same equipment and fluorochrome primers.

### Bayesian clustering analysis

A Bayesian clustering method [22] as implemented in the program STRUCTURE version 2.2 was used to infer the number of *K *unknown genetic populations in which the sampled multilocus genotypes can be split. This model-based Bayesian method also assigns a probability that the individuals belong to a certain population or to more than one population if they are admixed. In this analysis all 197 lineages were used (see Figure 1, Table 5). The data set was analysed using the admixture and uncorrelated allele frequencies models and *K *values 1–15 without incorporating population information. Four independent runs for each *K *were conducted with 100,000 iterations after a burn-in period of 20,000 iterations in each run.

### Allele frequencies, Hardy-Weinberg equilibrium and linkage disequilibrium

The sample of American and Far East aphids was not included in this analysis due to the low number of the individuals examined. In addition, the samples from Scotland (SCO) and England (ENO) were combined in one sample (UKO) as well as the European samples from tobacco (pooled sample are EUT). Therefore, seven populations were analysed. Allele frequencies, mean number of alleles per locus, observed (*H*_O_) and expected (*H*_E_) heterozygosity and inbreeding coefficient (*F*_IS_) were calculated using GENEPOP version 4.0 [60] (see also ). Allelic richness (*R*s = number of alleles independent of sample size) was also calculated using FSTAT version 2.9.3.2 [61], see also ). Differences in the average observed heterozygosity over all loci among populations were examined using the STRUC program of GENEPOP v. 3.4, which computes an unbiased estimate of the exact *P *value of a probability test of homogeneity on R × C contingency tables using a Markov chain method [62]. Deviation from Hardy-Weinberg (HW) equilibrium at each locus was examined separately using the U test [62] as implemented in GENEPOP version 4.0. A Markov chain (MC) method is used for the unbiased estimation of the exact *P *value of this test [63]. A multisample score test [62], which is performed by MC algorithm, was used as a global test across loci. Independence of microsatellite loci was examined with the G log-likelihood based exact test [64], which uses a simple modification of the MC algorithm described in Raymond & Rousset [62]. The latter two tests were performed using GENEPOP version 4.0.

### F_*ST *_and genetic distance analysis

Population structure was also assessed by calculating multilocus *F*_ST _values [65] for pairwise comparisons of samples using GENEPOP version 4.0. In addition, allelic differentiation between samples was examined using GENEPOP version 4.0. The test statistic is the G log-likelihood based exact test. In these analyses seven populations were used. To further investigate the genetic relationship between populations, a neighbour joining (NJ) tree based on the allele shared distance (DAS) [66] was constructed using the software POPULATIONS version 1.1.28 (). DAS distance counts the number of different alleles between multilocus genotypes. Bootstrap values were calculated by resampling loci, and are presented as percentages over 1000 replications.

## Authors' contributions

Microsatellite data were obtained and scored by LK, GM and BF. Statistical analyses were performed by JTM. The study was designed by all authors. The paper was written by JTM and BF. The manuscript has been read and approved by all authors.

## Supplementary Material

Additional file 1 **Posterior probability of the data estimated using Bayesian clustering analysis**. Posterior probability of the data (PPD) estimated using STRUCTURE against the number of *K *– clusters (a) and increase of PPD given *K *(b) calculated using the equation Ln (PPDK) – Ln (PPDK-1). Click here for file

Additional file 2 **Number and size of alleles**. Number of alleles and size ranges (in base pairs) of the six micosatellite loci examined. Click here for file
