# Supplementary figures and images for: Tracking the global dispersal of a cosmopolitan insect pest, the peach potato aphid
Source: BMC Ecol. 2009 May 11;9:13. doi: 10.1186/1472-6785-9-13 (PMC2687420; doi:10.1186/1472-6785-9-13)

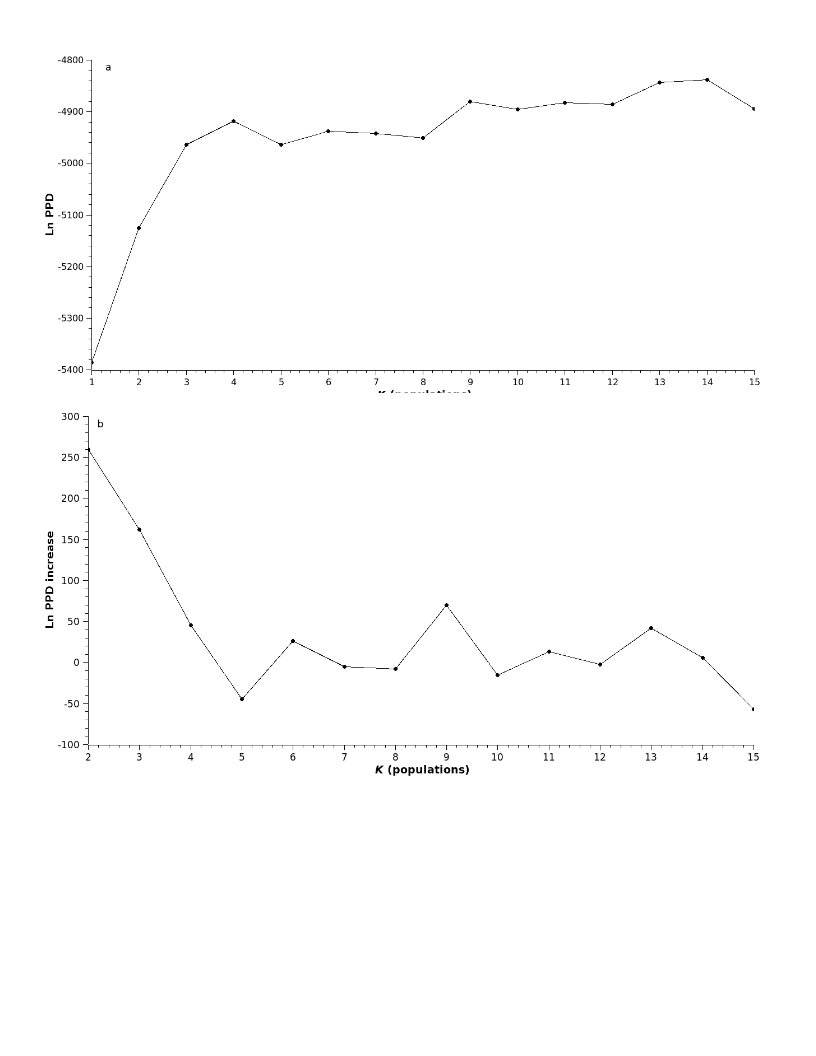

Supplement: Additional file 1 — Posterior probability of the data estimated using Bayesian clustering analysis. Posterior probability of the data (PPD) estimated using STRUCTURE against the number of K – clusters (a) and increase of PPD given K (b) calculated using the equation Ln (PPDK) – Ln (PPDK-1). [file 1472-6785-9-13-S1.tiff]
