# Supplementary material for: Tracking the global dispersal of a cosmopolitan insect pest, the peach potato aphid
Source: BMC Ecol. 2009 May 11;9:13. doi: 10.1186/1472-6785-9-13 (PMC2687420; doi:10.1186/1472-6785-9-13)
Supplement: Additional file 2 — Number and size of alleles. Number of alleles and size ranges (in base pairs) of the six micosatellite loci examined. [file 1472-6785-9-13-S2.pdf]

Locus	No. alleles	Range (in bp)	
		From	To
M35	12	178	204
M40	10	111	136
M49	33	114	213
M63	22	153	208
M86	21	95	161
myz9	16	196	228
